# Supplementary material for: Kaposi’s Sarcoma-associated herpesvirus uses a novel protein fold to hijack RNA Polymerase II for viral late gene transcription
Source: bioRxiv. 2026 Jun 29:2026.06.29.734287. Preprint. [Version 1] doi: 10.64898/2026.06.29.734287 (PMC13345323; doi:10.64898/2026.06.29.734287)
Supplement: Supplement 1 [file NIHPP2026.06.29.734287v1-supplement-1.pdf]

bioRxiv preprint doi: https://doi.org/10.64898/2026.06.29.734287; this version posted June 29, 2026. The copyright holder for this preprint (which was not certified by peer review) is the author/funder, who has granted bioRxiv a license to display the preprint in perpetuity. It is made available under a CC-BY 4.0 International license.

SUPPLEMENTARY FIGURES

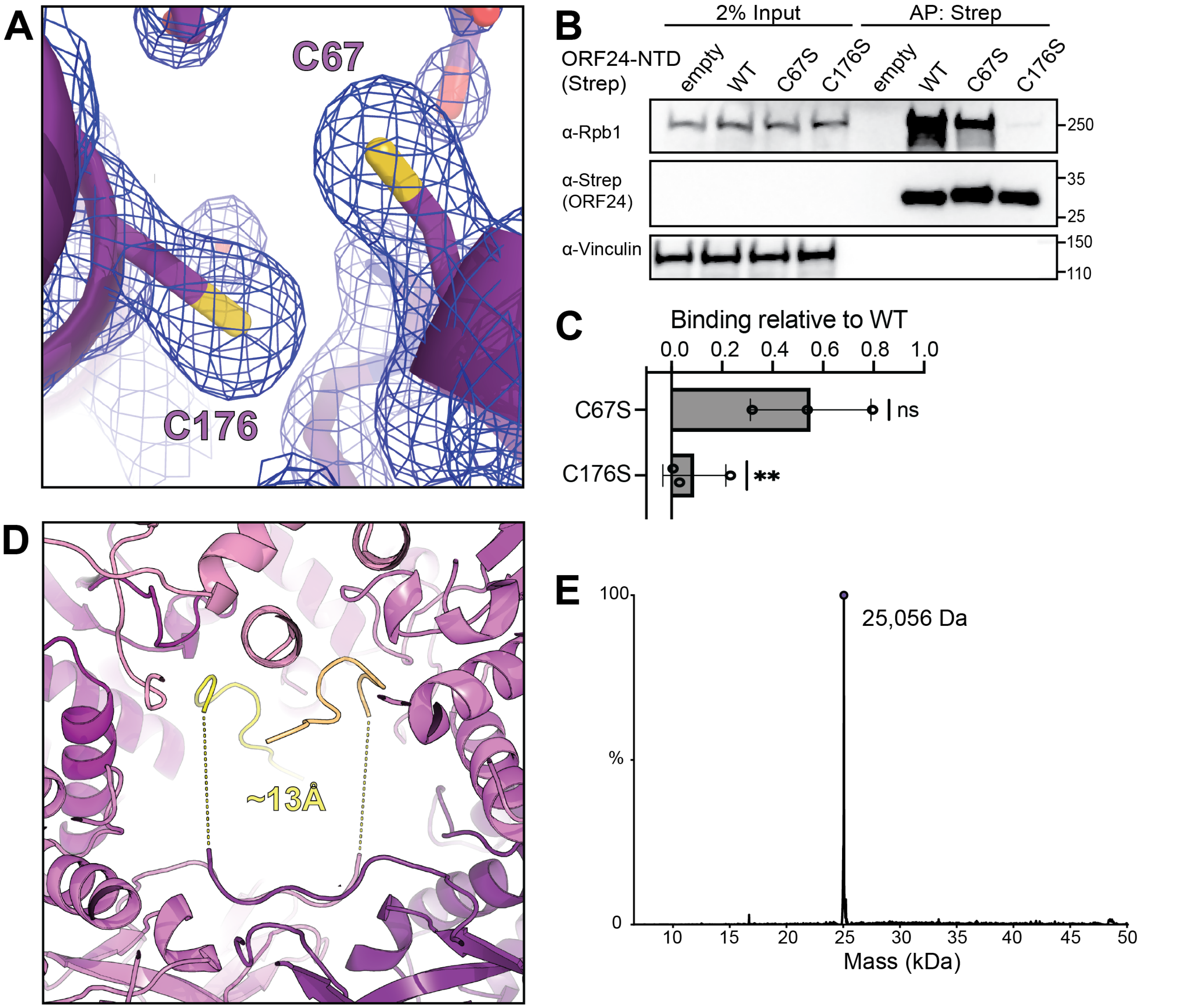

Supplementary Figure S1. Structural features of ORF24-NTD.
(A) *2mFo-DFc* electron density map of ORF24-NTD residues C67 and C176, contoured at 1σ. **(B)** Plasmids encoding wild-type or mutant strep-tagged ORF24-NTD were transiently transfected into HEK293T cells and subjected to affinity purification on StrepTactinXT beads followed by western blot analysis; representative of three biological replicates. Molecular weight (in kDa) was determined via a protein ladder and is indicated to the right. Vinculin serves as a loading control. **(C)** Quantification of three independent replicates of the affinity purification experiment shown in (B). The ratio of Rpb1 to ORF24-NTD was normalized to WT in each experiment. Data are from three biological replicates, with statistics calculated using a one sample t-test where WT binding was set to 1; $^{**}P < 0.01$. **(D)** The N-terminal hexahistidine tag may mediate crystal packing. The C-terminal ends of the modeled tag (SHHHHHHSSE) from two asymmetric units (yellow, wheat) are ~13 Å from the N-terminal (-1) Glycine. The intervening sequence (NLYFQ) is likely unstructured and spans this distance. ORF24-NTD is shown in shades of purple. **(E)** UPLC-MS/MS analysis of recombinantly purified His-ORF24-NTD was performed using a C4 BEH analytical column and reversed-phase gradient elution. The MS1 data was averaged across the 54 min elution profile and deconvoluted using UniDec to yield an average molecular weight measurement. The observed MS1 data correlates with an intact average mass of 25,056 Da, which closely matches the expected average molecular weight of unmodified His-ORF24-NTD minus the initiating Met (-131 Da). No other abundant ion signals were observed.

bioRxiv preprint doi: https://doi.org/10.64898/2026.06.29.734287; this version posted June 29, 2026. The copyright holder for this preprint (which was not certified by peer review) is the author/funder, who has granted bioRxiv a license to display the preprint in perpetuity. It is made available under a CC-BY 4.0 International license.

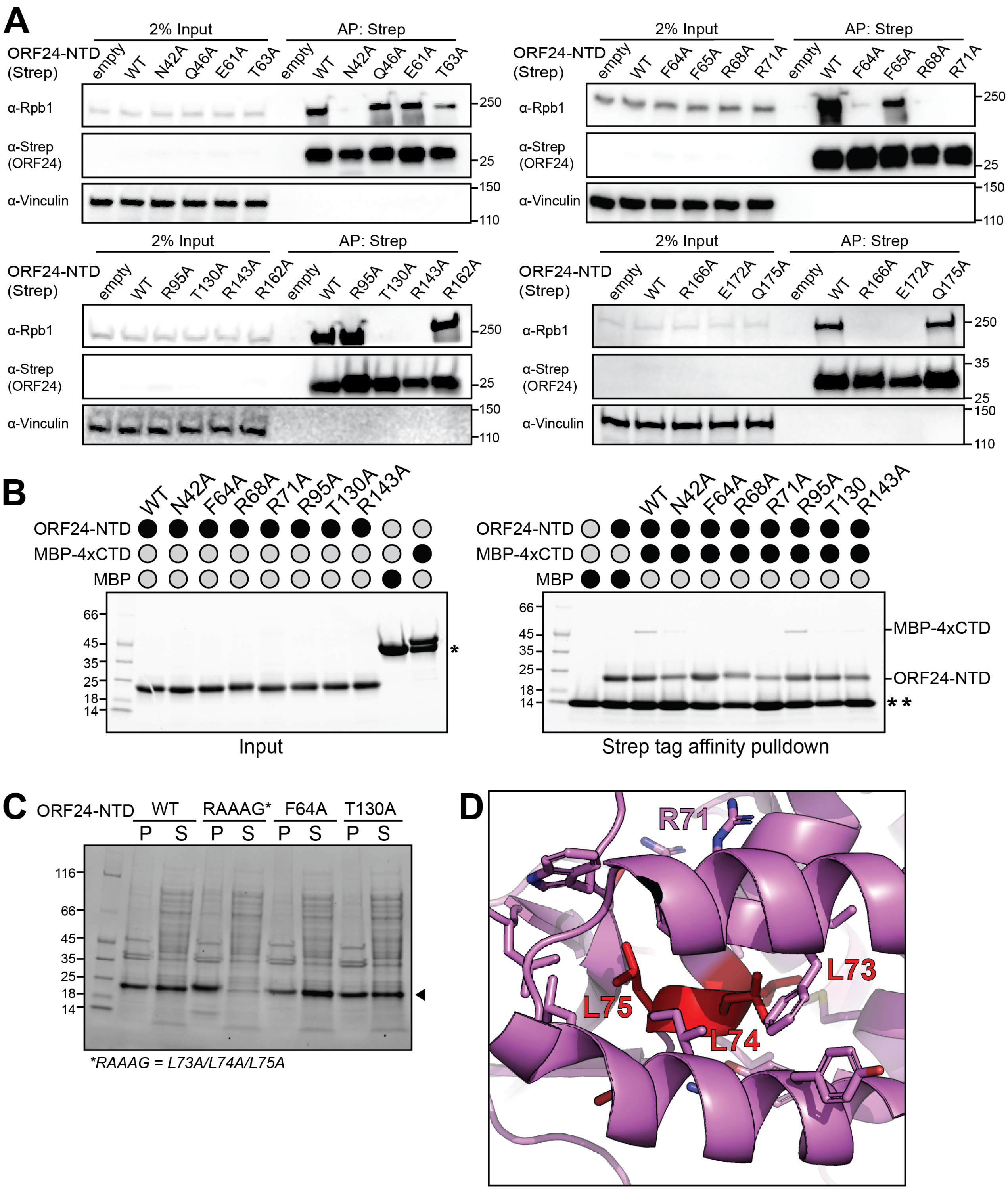

Supplementary Figure S2. Binding assays to monitor ORF24-NTD/RNAPII CTD interactions.
(A) Plasmids encoding wild-type or mutant strep-tagged ORF24-NTD were transiently transfected into HEK293T cells and subjected to affinity purification on StrepTactinXT beads followed by western blot analysis. Data are representative of three biological replicates. Molecular weight (in kDa) was determined via a protein ladder and is indicated to the right. Vinculin serves as a loading control. Data are quantified in **Figure 2B**. **(B)** Representative *in vitro* binding assay where recombinantly purified wild-type or mutant His-ORF24-NTD-strep was incubated with MBP-4xCTD followed by enrichment on StrepTactin XT beads. Eluted fractions were analyzed by stain-free SDS-PAGE. (*) indicates free MBP. (**) indicates a subunit of Strep-TactinXT released from the beads during boil elution. Data are quantified in **Figure 2D**. **(C)** Solubility analysis of His-ORF24-NTD mutants. Mutants were expressed in *E. coli* under identical conditions. After induction, equal amounts of cells were lysed, and the soluble and insoluble fractions were analyzed by stain-free SDS-PAGE. **(D)** Location of the “RAAAG” (L73A/L74A/L75A) mutation (red) on the ORF24-NTD structure (purple). These residues are buried in the interior of the protein between the base of the β-hairpin and α-helices α1 and α4-6.

bioRxiv preprint doi: https://doi.org/10.64898/2026.06.29.734287; this version posted June 29, 2026. The copyright holder for this preprint (which was not certified by peer review) is the author/funder, who has granted bioRxiv a license to display the preprint in perpetuity. It is made available under a CC-BY 4.0 International license.

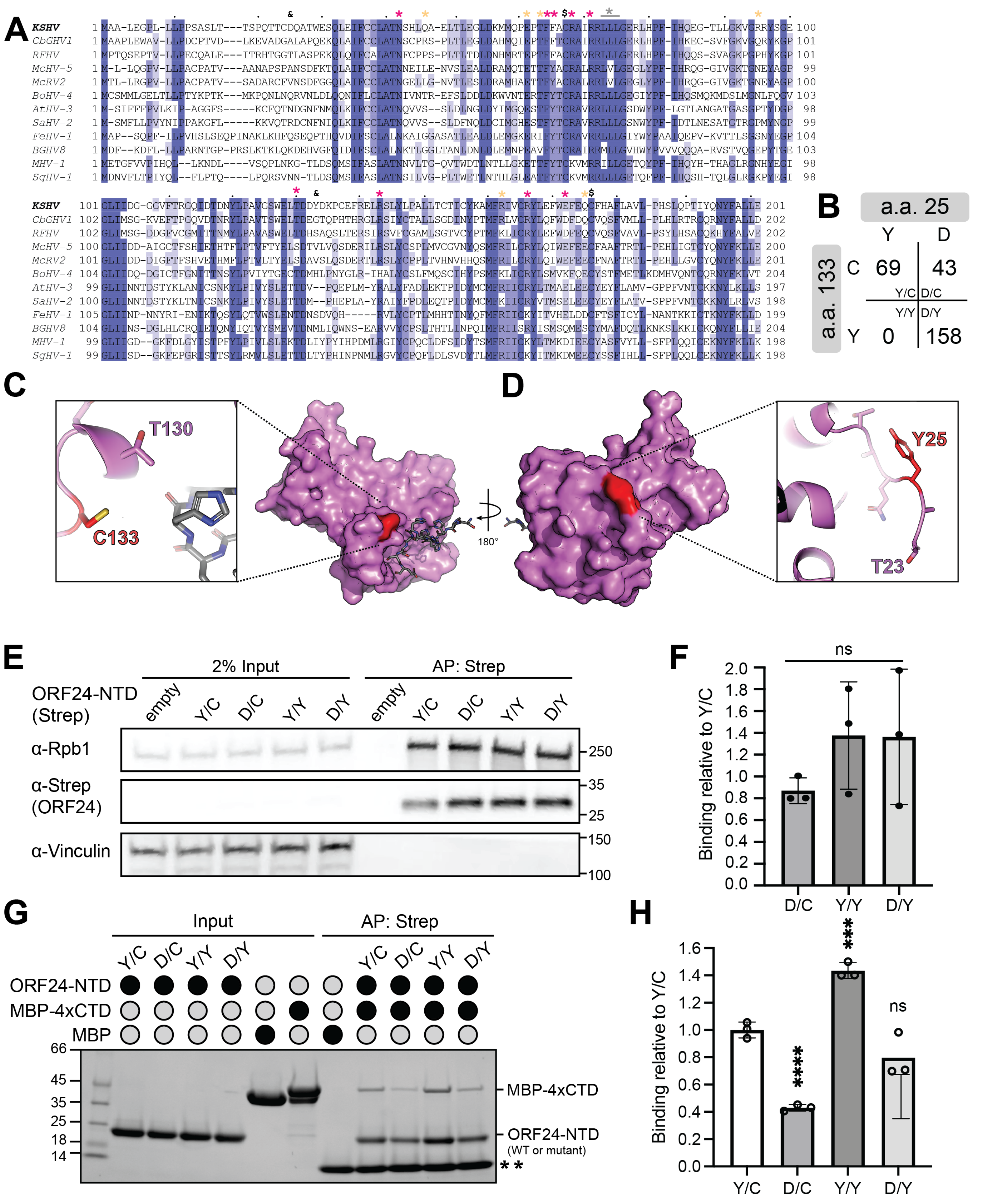

Supplementary Figure 3. Natural variation at ORF24 residues 25 and 133.
(A) Multiple sequence alignment of closely related gammaherpesvirus homologs of ORF24 where every tenth residue is indicated with a period above the alignment. Residues critical (pink) or not required for binding (yellow) as determined in Fig. 2 are indicated with asterisks. The grey asterisk marks site of the RAAAG mutations. Dollar signs indicate conserved cysteine residues with the potential to form a disulfide bond. Ampersands indicate positions 25 and 133 that are variable within sequenced KSHV genomes. **(B)** Distribution of naturally occurring residues present at positions 25 and 133 from

bioRxiv preprint doi: https://doi.org/10.64898/2026.06.29.734287; this version posted June 29, 2026. The copyright holder for this preprint (which was not certified by peer review) is the author/funder, who has granted bioRxiv a license to display the preprint in perpetuity. It is made available under a CC-BY 4.0 International license.

sequenced KSHV genomes available in NCBI. **(C)** Position and local contacts for residues C133 and **(D)** Y25 in the ORF24-NTD structure, highlighted in red sticks Nearby residues are shown in purple sticks, with the putative hexahistidine peptide shown in grey sticks. **(E)** Plasmids encoding ORF24-NTD harboring different combinations of residues at positions 25 and 133 were transiently transfected into HEK293T cells and subjected to affinity purification on StrepTactinXT beads followed by western blot analysis; representative of three biological replicates. Molecular weight (in kDa) was determined via a protein ladder and is indicated to the right. Vinculin serves as a loading control. **(F)** Quantification of three independent replicates of the affinity purification experiment shown in (E). The ratio of Rpb1 to ORF24-NTD was normalized to WT in each experiment. Data are from three biological replicates, with statistics calculated using a one sample t-test where WT binding was set to 1. **(G)** *In vitro* binding assay where recombinantly purified MBP-4xCTD and variants of His-ORF24-NTD-strep were enriched on StrepTactin XT beads. Free MBP was used as a negative control. (**) indicates a subunit of Strep-TactinXT released from the beads during boil elution. **(H)** Quantification of three independent replicates of the *in vitro* binding assay shown in (G). Data from three independent replicates were normalized to the average of wild-type binding, with statistics calculated using an unpaired t-test; ****P < 0.0001, ***P < 0.001.

bioRxiv preprint doi: https://doi.org/10.64898/2026.06.29.734287; this version posted June 29, 2026. The copyright holder for this preprint (which was not certified by peer review) is the author/funder, who has granted bioRxiv a license to display the preprint in perpetuity. It is made available under a CC-BY 4.0 International license.

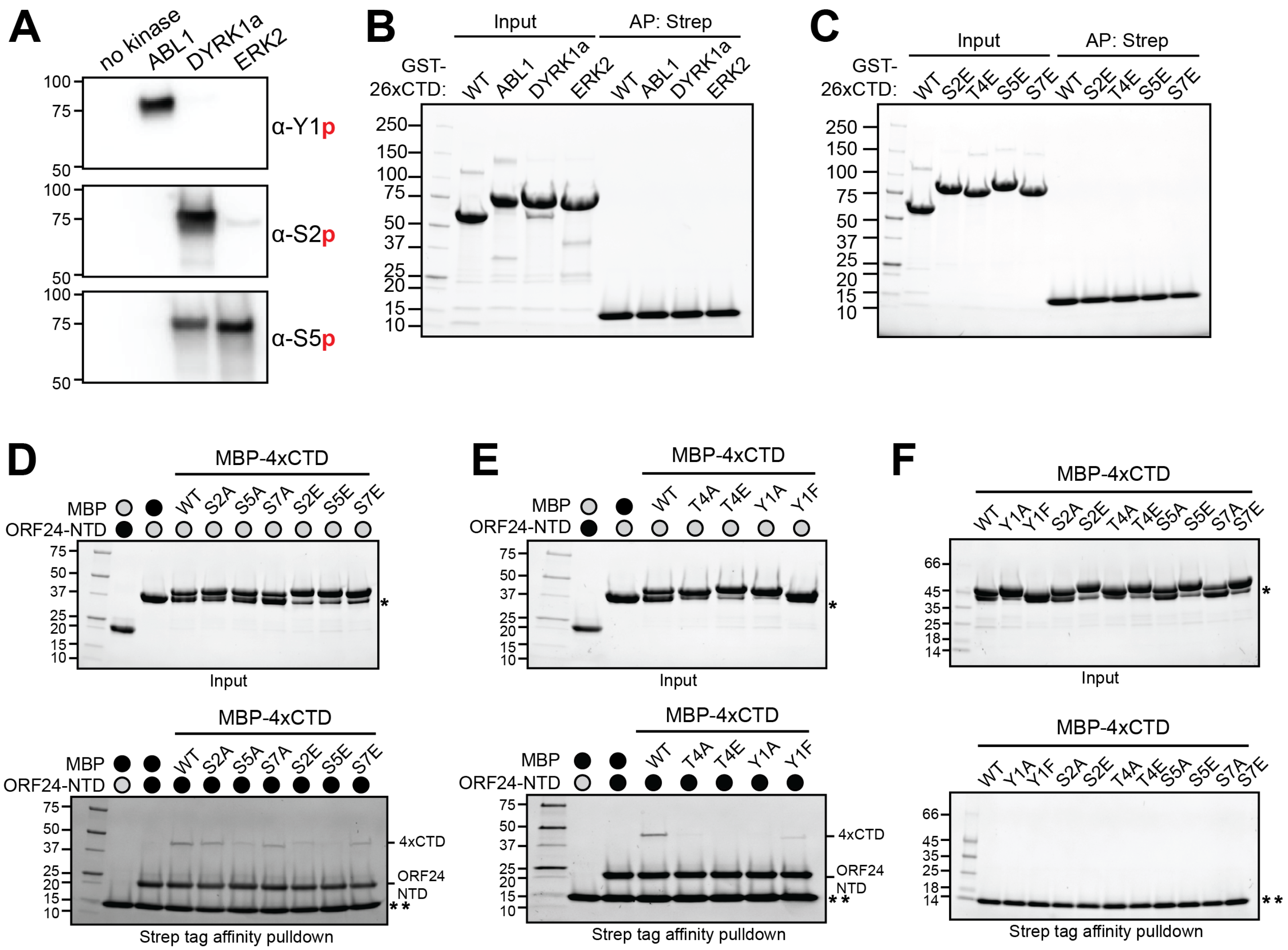

Supplementary Figure S4. Supporting data for phosphorylation of RNAPII CTD and mutant binding data. **(A)** Phosphorylated GST-26xCTD (0.2 µg) was analyzed by western blot with phospho-specific antibodies. **(B)** Phosphorylated GST-26xCTD or **(C)** GST-26xCTD phosphomimetic variants are not enriched by Strep-TactinXT beads in the absence of His-ORF24-NTD-strep. **(D)** Positions S2, S5, S7 and **(E)** Y1, T4 in the CTD heptad sequence affect binding of ORF24-NTD. Representative gels of an *in vitro* binding experiment quantified in Figure 4 using MBP-4xCTD mutants binding to His-ORF24-NTD-Strep. The top gel shows input (wild-type and mutant MBP-4xCTD and His-ORF24-NTD-strep) while the bottom gel shows the resulting enrichment from StrepTactinXT beads. (*) indicates free MBP, (**) indicates a subunit of Strep-TactinXT released from the beads during boil elution. Gels were visualized by stain-free imaging. **(F)** MBP-4xCTD variants do not bind StrepTactinXT beads in the absence of His-ORF24-NTD-strep. The top gel shows input (free MBP-4xCTD variants) and the bottom gel shows the resulting enrichment from StrepTactinXT beads.

bioRxiv preprint doi: https://doi.org/10.64898/2026.06.29.734287; this version posted June 29, 2026. The copyright holder for this preprint (which was not certified by peer review) is the author/funder, who has granted bioRxiv a license to display the preprint in perpetuity. It is made available under a CC-BY 4.0 International license.

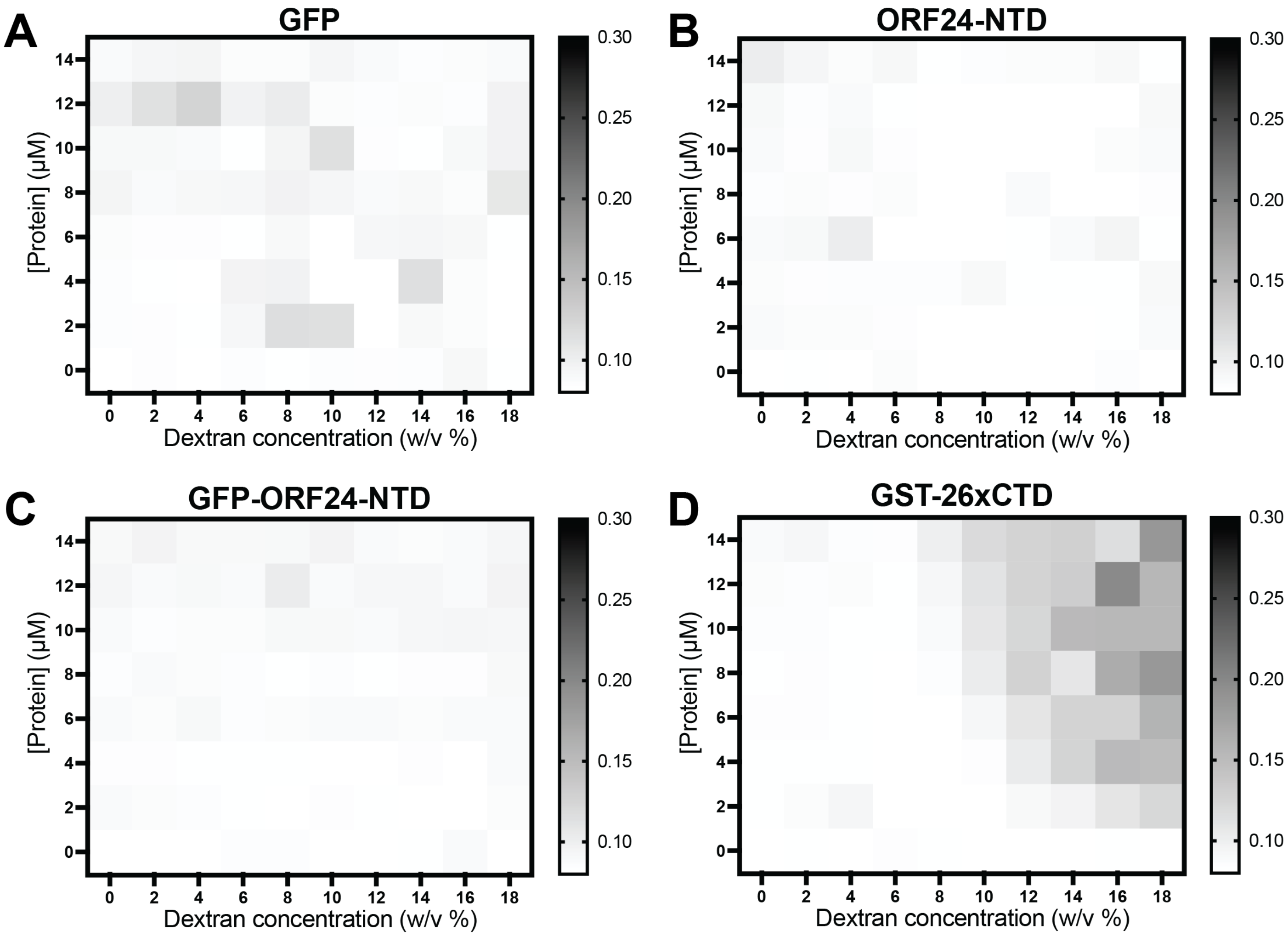

Supplementary Figure S5. RNAPII CTD, but not ORF24, phase separates *in vitro*.
Condensate formation at various concentrations of protein and dextran was monitored via absorbance at 350 nm for **(A)** free GFP, **(B)** ORF24-NTD-strep, **(C)** GFP-ORF24-NTD-strep, or **(D)** GST-26xCTD. Graphs represent the average of two independent replicates.

bioRxiv preprint doi: https://doi.org/10.64898/2026.06.29.734287; this version posted June 29, 2026. The copyright holder for this preprint (which was not certified by peer review) is the author/funder, who has granted bioRxiv a license to display the preprint in perpetuity. It is made available under a CC-BY 4.0 International license.

Supplementary Table 1 Crystallographic data collection, phasing and structure refinement statistics.

**Parameter**	**ORF24-NTD (PDB 13DH)**
**Data Collection**	
Wavelength (Å)	0.9795
Resolution range (Å)	36.75 - 1.932 (2.08 - 1.93)
Space group	P 41 21 2
Unit cell	
a, b, c (Å)	51.97, 51.97, 147.6
α=β=γ (°)	90
Total reflections	227,717
Unique reflections	13,138 (1,143)
Multiplicity	17.3
Completeness (%)	82.39 (36.82)
Mean I/sigma(I)	17.8
Wilson B-factor	38.86
R-merge	10.50%
R-meas	10.80%
CC1/2	12.1
CC*	99.9
**Refinement Statistics**	
Resolution range (Å)	36.75 - 2.04 (2.04 - 1.93)
R-work / R-free	0.1860 / 0.2434 (0.3474/0.3684)
Number of non-hydrogen atoms	1891
macromolecules	1713
ligands	60
solvent	118
Protein residues	213
RMS(bonds)	0.011
RMS(angles)	1.76
Ramachandran favored (%)	96.17
Ramachandran allowed (%)	3.35
Ramachandran outliers (%)	0.48
Rotamer outliers (%)	6.45
Clashscore	3.42
Average B-factor	58.11
macromolecules	56.95
ligands	87.36
solvent	60
Number of TLS groups	2 (for protein and peptide)
